# Measuring Blink-Related Brainwaves Using Low-Density Electroencephalography with Textile Electrodes for Real-World Applications

**DOI:** 10.3390/s25144486

**Published:** 2025-07-18

**Authors:** Emily Acampora, Sujoy Ghosh Hajra, Careesa Chang Liu

**Affiliations:** Department of Biomedical Engineering and Science, Florida Institute of Technology, 150 W University Blvd, Melbourne, FL 32901, USA

**Keywords:** blink-related oscillations (BRO), electroencephalography (EEG), textile electrode, resting state, brain function assessment, blinking

## Abstract

**Background**: Electroencephalography (EEG) systems based on textile electrodes are increasingly being developed to address the need for more wearable sensor systems for brain function monitoring. Blink-related oscillations (BROs) are a new measure of brain function that corresponds to brainwave responses occurring after spontaneous blinking, and indexes neural processes as the brain evaluates new visual information appearing after eye re-opening. Prior studies have reported BRO utility as both a clinical and non-clinical biomarker of cognition, but no study has demonstrated BRO measurement using textile-based EEG devices that facilitate user comfort for real-world applications. **Methods**: We investigated BRO measurement using a four-channel EEG system with textile electrodes by extracting BRO responses using existing, publicly available EEG data (*n* = 9). We compared BRO effects derived from textile-based electrodes with those from standard dry Ag/Ag-Cl electrodes collected at the same locations (i.e., Fp1, Fp2, F7, F8) and using the same EEG amplifier. **Results**: Results showed that BRO effects measured using textile electrodes exhibited similar features in both time and frequency domains compared to dry Ag/Ag-Cl electrodes. Data from both technologies also showed similar performance in artifact removal and signal capture. **Conclusions**: These findings provide the first demonstration of successful BRO signal capture using four-channel EEG with textile electrodes, providing compelling evidence toward the development of a comfortable and user-friendly EEG technology that uses the simple activity of blinking for objective brain function assessment in a variety of settings.

## 1. Introduction

Spontaneous blinking is a simple activity that has recently shown promise as a potential avenue for evaluating cognition. Studies examining brainwave responses occurring following blinking have demonstrated neural phenomena known as blink-related oscillations (BROs), which index environmental processing and awareness responses as the brain evaluates new images appearing when the eyes re-open following a blink [1,2]. BRO responses have been shown to engage the precuneus brain regions known to be involved in higher-level cognitive processes, such as visuo-spatial processing [3,4,5], episodic memory [6,7,8], and self-related processing [9,10,11]. The associated neurocognitive effects have also been shown to encompass a wide range of neural mechanisms, including sensory processing, episodic memory, and information processing [12]. Previous studies have demonstrated BRO responses in healthy adults across a variety of task states, including resting [1], cognitive loading [12], and different sensory environments [13]. The associated BRO effects have been shown to be modulated by the underlying cognitive state of the brain at the time the blink occurred, as spontaneous blinking during the performance of a challenging cognitive task leads to reduced extent of BRO-induced cortical activations compared to blinking during rest [12]. Additionally, BRO effects are also modulated by the ongoing sensory environment at the time blinking occurred, as blinking while exposed to ongoing visual inputs results in decreased amplitude of BRO responses compared to blinking at rest, or when the same inputs are presented in auditory format [13]. BRO responses have also been reported under other task states such as n-back working memory [14], as well as complex, multi-domain tasks such as the multi-attribute task battery (MATB) [15]. These findings suggest that, in addition to providing information about brain responses to blinking, the BRO phenomenon may also be leveraged as a potential tool for evaluating brain function and cognitive status.

Recent studies have demonstrated the efficacy of BRO responses in assessing cognitive performance in real-world scenarios, as BRO response amplitudes have been shown to detect workload differences due to task difficulty in pilots performing simulated flight maneuvers using both a computerized flight task [15] as well as a high-fidelity flight simulator [16]. Additionally, the BRO response characteristics were also sensitive in detecting age-related effects in flight task difficulty when comparing older vs. younger pilots [16]. In addition, other studies have also demonstrated that BRO responses may be utilized for the clinical assessment of brain function changes in different populations, as BRO response characteristics have been shown to reflect aging-related brain changes in cognitively normal healthy adults—with greater sensitivity compared to behavior-based measures such as reaction time and accuracy of task performance [17]. Moreover, BRO effects have also been shown to detect brain function changes in soccer athletes after they experience subconcussive head impacts due to headers in regular play—despite none of the athletes having any diagnosis of concussion [18]. Together, these studies provide promising evidence towards the potential utility of BRO responses as a diagnostic tool for brain function evaluation across a variety of scenarios.

In order to develop a BRO-based technology that can be widely used for brain function monitoring in everyday life, hardware technology platforms are required that are portable, easy to use, comfortable to wear, and allow for rapid application. However, this is currently not possible with existing BRO measurements, as prior studies have all employed either large, fixed-infrastructure devices like magnetoencephalography (MEG) [1,12], or utilized high-density 64-channel electroencephalography (EEG) systems with full-head coverage [13,19]. A previous study had developed software analytic techniques to extract BRO responses from only four sensors [19], but the data in that study were still acquired by a 64-channel research-grade EEG system. No study to date has demonstrated the ability to measure BRO responses using data directly obtained from low-density EEG systems with less than five electrodes. Additionally, prior EEG-based BRO studies have also employed wet-electrode EEG systems that utilized silver–silver chloride (Ag/AgCl) electrodes with electrolytic gel to increase conductivity, which enhances signal quality but requires long preparation times for data collection. In contrast, a portable BRO solution that enables rapid application should ideally employ EEG hardware that is easy to use and comfortable to wear, without the need for conductive gel.

Textiles can be used to manufacture conductive materials for electrodes via several different approaches, such as silver-coated polymers [20], conductive polymers [21,22,23], and graphene [24]. Relative to traditional Ag/AgCl electrodes, textile-based electrodes hold several key advantages that make them ideal candidates for creating user-friendly BRO measurement devices for everyday wear. For instance, textile-based electrodes do not require the use of electrolytic gel, which reduces set-up time and allows for easy application and improved user-friendliness. In addition, textile-based electrodes are light, breathable, and flexible, making them comfortable to wear for the user [25,26]. Textile-based electrodes can also be integrated into fabrics such as clothing, thus facilitating repeated measurements in daily life with minimal inconvenience [27]. Finally, textile electrodes can be easily manufactured, which reduces the cost of fabrication and enhances their accessibility, making them well-suited for large-scale production [27]. While textile-based electrodes have primarily been used for electrocardiography (ECG) applications in the past [28,29], recent developments have also shown increasing interest in utilizing textile-based electrodes for EEG systems [30]. One study reported a textile-based EEG monitoring system for neonatal intensive care units (ICUs), designed to enable long-term monitoring of newborn infants using electrodes that minimize skin irritation [31]. Other studies have reported portable, point-of-care EEG systems using graphene and metal-plated textile-based electrodes incorporated into a simple headband design [27,32]. Together, these prior findings point to the potential utility of textile-based electrodes for portable EEG-based brain function assessments.

In this study, we undertook the first investigation of BRO measurement using portable, low-density EEG systems with four channels. Specifically, we compared BRO responses measured using two EEG systems containing the same amplifier hardware, but using different electrode materials: one system utilized textile-based electrodes, while the other used dry Ag/AgCl electrodes. Our results demonstrate for the first time that BRO brain responses can be successfully captured using only four sensors, and both dry Ag/Ag-Cl and textile-based EEG systems were able to detect salient BRO characteristics. Moreover, both the dry and textile EEG systems exhibited comparable performance in capturing BRO responses.

## 2. Materials and Methods

### 2.1. Participants

This study used publicly available EEG data from López-Larraz et al. [27]. The participant sample consisted of 10 healthy adult volunteers (5 females, 5 males; age: 27.8 ± 3.7 years). Each participant provided written informed consent prior to data collection. Ethical approval was obtained from the Institutional Review Board of the Florida Institute of Technology for the secondary analysis of existing human physiological data (Protocol # 24-177).

### 2.2. Data Acquisition 

**Figure 1:**
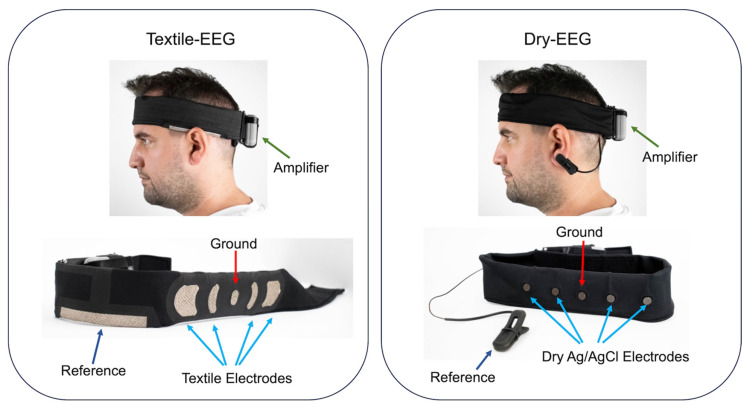
Images of the form factors for the Textile-EEG and Dry-EEG systems, showing the reference and ground electrodes as well as recording electrodes corresponding to F7, Fp1, Fp2, and F8 locations. Adapted from López-Larraz et al. [27].

Data collection has been described in detail elsewhere [27]. Briefly, three minutes of eyes-open resting-state data were collected using each of the textile-based EEG (Textile-EEG) and dry-electrode EEG (Dry-EEG) systems, with the order counter-balanced across participants. Data were sampled at 256 Hz. Images of the two systems are shown in Figure 1. Both systems had 4 recording electrodes in the same locations across the forehead (F7, Fp1, Fp2, and F8), chosen to increase surface contact at the skin–electrode interface. The ground electrodes for both systems were placed at Fpz. The Textile-EEG system employed the reference electrode positioned at the upper union between the left ear and the head, while the Dry-EEG system utilized a reference electrode in the form of an ear clip located on the left ear.

Details of the Textile-EEG system have previously been described elsewhere [27]. Briefly, the textile electrodes were woven using 3-stranded, silver-coated Nylon forming a conductive yarn, with a linear resistance of 114 Ω/m. Electrode surface areas were 2.8 cm^2^ for Fp1 and Fp2 electrode locations, and 7.4 cm^2^ for the F7 and F8 to accommodate different head sizes. The headband was created using combinations of different textile materials, including polyamide, polyurethane, polyester, elastomer, and Nylon. Textile fiber paths for signal transmission were created by overlapping materials across different textile layers to emulate the effect of coaxial cables. The ‘wire’ conductor was created by embroidering using 8-stranded silver-coated Nylon yarns, then insulating the conductor using vinyl, heat-sealing, and further shielding by adding another conductive layer made using Nylon coated with silver, copper, and tin. The overall structure was then covered using Nylon to isolate and affix the transmission lines to the fabric. The impedance of the textile electrode has previously been characterized as 368 kΩ and 171 kΩ at 1 Hz and 10 Hz, respectively [27].

The Dry-EEG system employed conventional Ag/AgCl electrodes configured in a similar headband format, with the electrodes having a circular shape and a diameter of 0.8 cm [27]. The impedance of the dry Ag/AgCl electrode was also previously characterized to be 110 kΩ and 78 kΩ at 1 Hz and 10 Hz, respectively [27].

**Figure 2:**
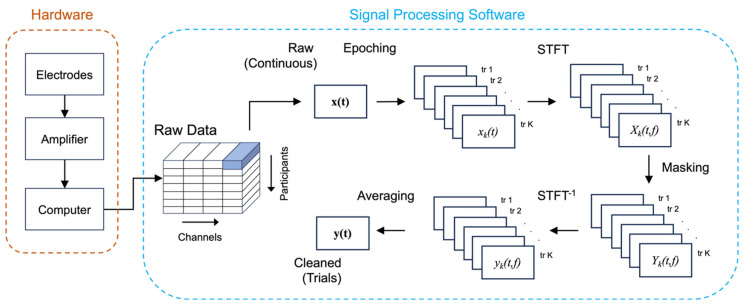
Schematic overview of the data collection hardware and signal processing software. (**Left panel**): The hardware components comprise front-end textile or dry electrodes connecting to the amplifier, which then transmits the data via Bluetooth to a computer for storage. (**Right panel**): The raw data are processed to remove artifact using the TF filter technique. Raw continuous data for each channel are first segmented into epochs according to identified blink instances, then transformed into the frequency domain using STFT, masked to remove artifact, inverse-transformed back to the time domain, and averaged across trials to derive the cleaned trial-level data.

The EEG amplifier hardware consisted of EEG-Hero^TM^ (Bitbrain, Bitburg, Spain), with 50 GΩ input impedance and 100 dB common mode rejection ratio [27]. This amplifier had previously been benchmarked against medical-grade wet-electrode EEG systems, and demonstrated high fidelity in brain computer interface applications [33]. A schematic overview of the data collection hardware is shown in Figure 2.

### 2.3. Preprocessing and Blink Detection

All analyses for this study were undertaken using Matlab R2022b. Each participant’s data were first visually inspected to determine the presence of large amplitude noise, and none were found. The data were notch-filtered at 50 Hz to remove power line noise, then band-pass filtered to 0.5–20 Hz frequency using a zero-phase, 4th-order butterworth filter. Blink identification was subsequently performed using a convolution-based, semi-automated, template matching procedure as described in previous studies [1,12]. Briefly, the Fp1 electrode was chosen as the vertical electrooculogram (vEOG) channel due to its proximity to the eye, and data were band-pass filtered to 0.5–16 Hz. A stereotypical blink instance was then manually selected as ‘template’, and this template was convolved with the entire vEOG signal. Candidate blink instances were then identified by applying an amplitude threshold to select blink events sufficiently similar to the template. Thereafter, a temporal threshold was also applied to exclude blink events that are less than 3 s apart in order to minimize contamination from adjacent blinks. This procedure was performed separately for each participant and dataset. Data from one of the ten participants were excluded from subsequent analyses due to the absence of identifiable blink events. The final dataset for BRO signal extraction thus comprised a total of nine participants.

### 2.4. Data Denoising

Data denoising was performed using a time-frequency filtering (TF filtering) technique as previously described [19], and the procedure is illustrated in Figure 2. Briefly, each participant’s raw continuous data were segmented into 3-s epochs with the blink maximum centered at time zero or *T*_0_. Time-frequency transformation was then applied to each epoch and electrode using Short-Time Fourier Transform (STFT). This is performed for each channel *i* = 1…*C* and trial *k* = 1…*K* as follows:

(1) $$X_{i,k}\left(t,f\right)=\int_{-\infty }^{\infty }x_{i,k}\left(\tau \right)w\left(t-\tau \right)e^{-j2\pi f\tau }$$

where $x_{i,k}(t)$ is the epoched raw data, $w\left(t\right)$ is a 128 ms Hamming window with a 100 ms hop length, and $X_{i,k}(t,f)$ is the corresponding data in the time-frequency domain. A binary mask *M*(*t*,*f*) was then constructed by applying a unity gain for time-frequency features corresponding to BRO signal, and a gain of zero for all other time-frequency locations. The piece-wise function for the binary mask was defined as follows:

(2) $$M\left(t,f\right)=\left\{\begin{array}{c} 0,\\ 0,\\ 1, \end{array}\begin{array}{c} \;\;\;\;\;\;\;f<0.5;\;f>4.0\;\\ \;\;\;\;\;\;-0.5<t<0.1\;\;\\ otherwise \end{array}\right.$$

where *t* is time in seconds and *f* is frequency in Hz. The parameters of the binary mask were selected based on prior literature [19], and the same mask was used for both technologies and across all participants. The mask was then applied to the STFT-transformed signal in the frequency domain for each trial:

(3) $$Y_{i,k}\left(t,f\right)=X_{i,k}\left(t,f\right)\cdot M(t,f)$$

Thereafter, inverse STFT was applied to transform the masked signal back to the time domain, and the masked trials were baseline-corrected by subtracting from each trial the mean signal from the pre-blink interval (−1500, −500) ms and averaged together to obtain the cleaned BRO signal for each electrode:

(4) $$y_{i,k}\left(t\right)=\int_{-\infty }^{\infty }Y_{i,k}\left(t,f\right)e^{j2\pi f\tau }$$

(5) $$y_{i}\left(t\right)=\frac{1}{K}\sum_{k=1}^{K}\left(y_{i,k}\left(t\right)-\bar{y_{ik}}\right)$$

where $y_{i,k}\left(t\right)$ is the time-domain signal for the *i*th channel and *k*th trial, $\bar{y_{i,k}}$ the mean amplitude within the pre-blink baseline interval in the corresponding trial, $y_{i}\left(t\right)$ is the trial-averaged cleaned signal, and K is the total number of trials. The same procedure was undertaken for both the Textile-EEG and Dry-EEG systems.

### 2.5. Performance Evaluation

Outcome measurements were computed using the cleaned BRO signal to evaluate the performance of the Textile-EEG and the Dry-EEG systems. Evaluations included assessment of the effectiveness of artifact removal, comparison of signal morphology, and examination of the ability of each EEG technology to capture salient BRO response characteristics in both time and frequency domains.

#### 2.5.1. Effectiveness of Artifact Removal

The effectiveness of ocular artifact removal was assessed both qualitatively and quantitatively. Qualitative assessment involved comparing individual-level, trial-averaged data before and after artifact removal to ensure the elimination of temporal and spectral features consistent with ocular artifact, such as large positive spike at blink latency or *T*_0_ in the time domain, as well as high signal power in the delta-band at *T*_0_ in the frequency domain [19]. Quantitative assessment of artifact removal utilized ocular contamination index (OCI) as described in previous literature [1,13,19]:

$$OCI=\left|\frac{y_{i}}{x_{i}}\right|$$

where *y_i_* and *x_i_* are the trial-averaged signal amplitudes at 0 ms (blink maximum) and −1000 ms (pre-blink baseline), respectively. OCI measures the ratio of signal power contribution at blink latency (time 0 or *T*_0_) relative to that in the pre-blink baseline (−1000 ms). Results were compared between the cleaned and raw data using the Wilcoxon signed rank test.

#### 2.5.2. Time-Domain BRO Response and Signal Capture

BRO response characteristics were evaluated using the cleaned data in both the time and frequency domains to examine the performance of the two EEG systems. Time-domain characteristics were assessed by averaging across participants the individual-level, trial-averaged cleaned BRO waveforms to derive the grand-averaged BRO responses for each channel. Salient BRO characteristics were measured by identifying two BRO component features as the largest peaks within pre-defined intervals using individual-level trial-averaged waveforms in accordance with prior literature [13]. In particular, the first component (C1) was identified as the largest positive peak within the (0–190) ms window, while the second component (C2) was defined as the largest negative peak within the (210–450) ms interval. The peak amplitudes of the C1 and C2 components were computed to be the mean amplitude within a 20 ms window spanning the corresponding peaks, and peak latencies were determined to be the corresponding time of each component peak. The baseline amplitude was computed as the mean signal amplitude within the (−1300, −1100) ms pre-blink interval. Results for both amplitude and latency were statistically compared between the Textile-EEG and Dry-EEG systems for each electrode using a paired *t*-test, with *p* < 0.05 as threshold for significance. Additionally, Pearson correlation was also used to compare the amplitudes and latencies between the two technologies to determine the consistency of measurement across device platforms, with *t*-test to assess for significant deviation of the correlation coefficient from zero.

#### 2.5.3. Frequency-Domain BRO Response

Frequency-domain BRO response characteristics were extracted by applying a continuous wavelet transform (CWT) with Morlet function and 6 cycles in line with prior literature [12]. This was carried out at the individual level for each channel and trial, and the logarithm of the squared absolute values of the wavelet coefficients was computed to derive the log spectral power. To determine blink-related effects, baseline correction was performed by subtracting from each trial the mean log spectral power during a pre-blink interval ranging from −1500 ms to −500 ms [1,13]. Results were averaged across trials for each subject and channel, then averaged across all participants to derive the grand-averaged time-frequency characteristics. Analyses were performed separately for each of the two EEG systems. Statistical comparison between the Textile-EEG and Dry-EEG systems was undertaken using a permutation-based approach adapted from Monte Carlo estimates, in line with prior literature [12,13,34]. In particular, the log spectral power for each time point and frequency was first compared between the two EEG systems using paired *t*-test to derive a group-level T-statistic value. The spectral power was then randomly permuted across participants and EEG systems, and a permuted T-statistic value was generated. This permutation was repeated 1000 times to create a null distribution of permuted T-statistic values, and the true T-statistic was compared with this null distribution to determine probabilities. Results were deemed significant if *p* < 0.05.

#### 2.5.4. Morphological Comparisons

Morphological evaluation in the time domain was performed by examining the consistency between the Dry-EEG and Textile-EEG waveforms using intraclass correlation (ICC), in line with previous studies [19,35]. In particular, ICC values were computed at the individual level between the two technologies using trial-averaged waveforms, and statistical significance was assessed with a nonparametric permutation approach adapted from Monte Carlo estimates [19,36]. Specifically, ICC values were generated by first randomly permuting the data across all subjects and technologies, then re-computing ICC values at the individual level before averaging across subjects. This process was repeated 1000 times to create a permuted null distribution of ICC values, and the probability of significance was then determined by comparing the true ICC value with the null distribution. Probabilities below 0.05 were considered to be significant. 

Morphological evaluation in the frequency domain was performed in a similar manner to that in the time domain, with minor modifications. Spectral power coefficients were first binned into 3 different frequency bands corresponding to low delta (0.5–1.5 Hz), mid-delta (1.5–3.5 Hz), and high delta (3.5–4.5 Hz). The spectral coefficients were then summed across frequencies for each participant in order to generate a time-evolved series of spectral coefficients for each of the 3 frequency bins. Individual-level ICC was computed between the Dry-EEG and Textile-EEG during the (0–1000) ms post-blink interval for each frequency bin, and results were averaged across subjects. Statistical evaluation was performed in a similar fashion to the time-domain results earlier, using 1000 random permutations to generate a null distribution for each frequency bin. Probabilities below 0.05 were deemed to be significant.

## 3. Results

### 3.1. Effectiveness of Artifact Removal

**Figure 3:**
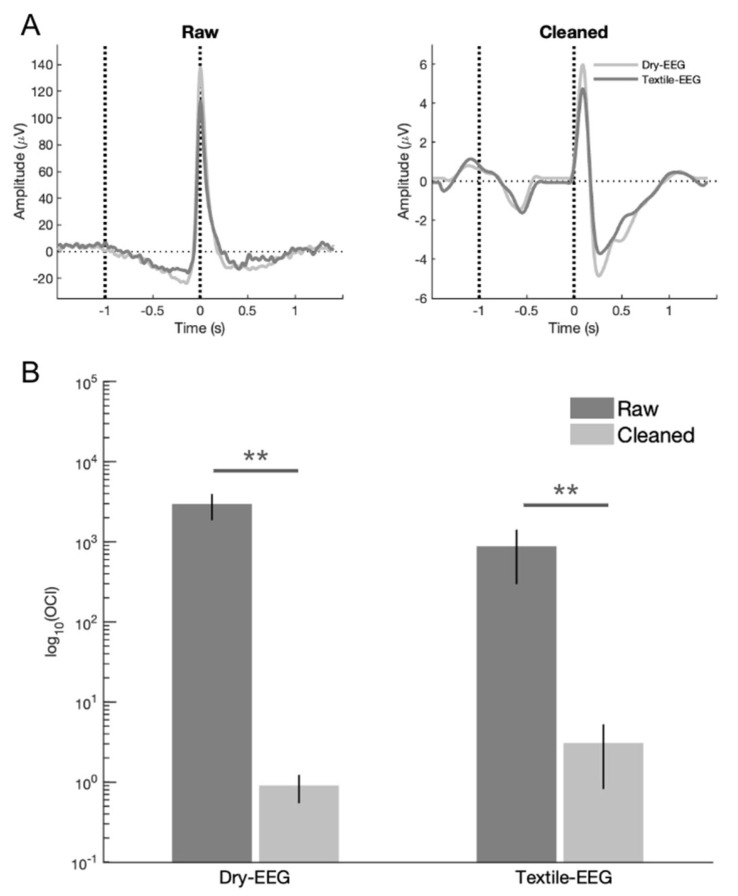
(**A**) Representative subject data for both EEG systems showing the disappearance of ocular artifact in the form of large signal spike at time 0 after data cleaning. Black dotted line at time 0 denotes latency of maximum blink amplitude or *T*_0_; black dotted line at –1000 ms corresponds to pre-blink baseline. (**B**) Ocular contamination index (OCI) results showing the ratio of signal power contribution at blink latency relative to that during the pre-blink baseline for both EEG systems. OCI is significantly reduced by more than 99.5% after data cleaning for both technologies. ** *p* < 0.001.

To determine the effectiveness of artifact removal, qualitative and quantitative comparisons were undertaken to compare signal characteristics before and after data cleaning. Qualitative assessment showed that signal features consistent with ocular artifact, such as large signal spike at blink latency (time 0) in the time domain and high signal power at time 0 in the frequency domain, were successfully removed after data cleaning for both the Textile-EEG and Dry-EEG systems (Figure 3A). In addition, quantitative assessment using OCI showed significant reduction in ocular signal contribution for both the Textile-EEG and Dry-EEG following data cleaning (*p* < 0.001, Figure 3B), with both technologies exhibiting greater than 99.5% reduction in ocular artifact. There were no significant differences in OCI between the Dry-EEG and Textile-EEG systems in either raw ($t_{8}$ = 1.72, *p* = 0.12) or cleaned ($t_{8}$ = −0.96, *p* = 0.36) data, indicating that both technologies showed comparable extent of artifact removal.

### 3.2. BRO Response Characteristics

**Figure 4:**
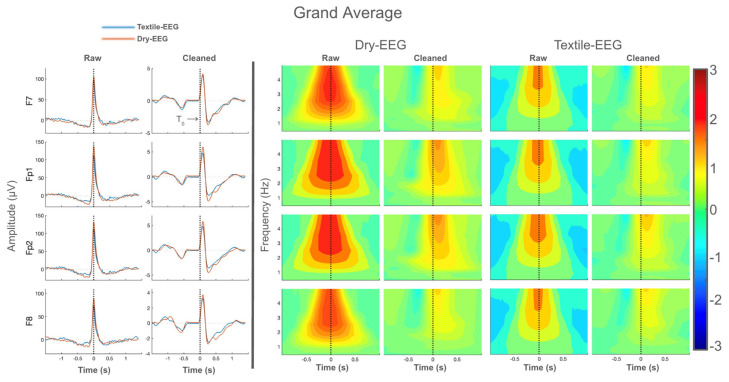
Grand-averaged results for all four electrodes for both technologies, showing both raw and cleaned data. (**Left panel**): Time-domain results showing large signal spike corresponding to ocular artifact at blink latency in the raw data, which disappears after data cleaning. Black line denotes the latency of maximum blink amplitude or *T*_0_. (**Right panel**): Frequency-domain results showing high signal power concentrated at blink latency in the raw data, which disappears after cleaning. Color bar represents log power values.

Grand-averaged time-domain waveforms for both technologies are shown in Figure 4. Large signal spikes consistent with ocular artifact are present at blink latency (time 0 or *T*_0_) in the raw data for all electrodes for both EEG systems, but these signal features disappeared following artifact removal (Figure 4, left panel). Instead, cleaned data exhibited positive and negative peaks located at approximately 100 ms and 250 ms, respectively, in line with known features of the BRO response [1,19]. Similarly, grand-averaged frequency-domain results also showed spectral power concentrated at blink latency in the raw data, consistent with ocular artifact (Figure 4, right panel). However, this disappears after data cleaning for all electrodes in both technologies, and the peak spectral power shifts to ~200 ms, also consistent with known characteristics of the BRO response [19]. These results suggest that both the Dry-EEG and Textile-EEG systems captured BRO responses in line with prior literature.

### 3.3. Signal Capture in Time Domain

**Figure 5:**
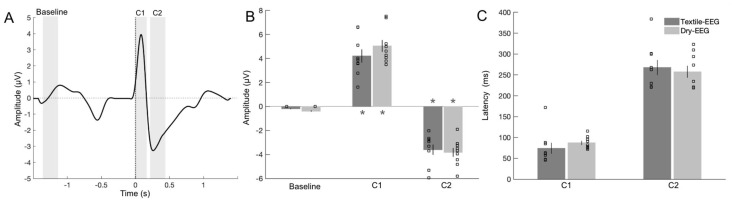
Results for time-domain signal capture in the Textile-EEG and Dry-EEG systems. (**A**) Grand-averaged BRO waveform for the Fp1 channel. Highlighted intervals show windows of interest used to compute signal amplitudes corresponding to the C1 and C2 components, as well as the pre-blink baseline. (**B**) BRO component amplitudes. * *p* < 0.01 compared to baseline. (**C**) BRO component latencies. Results are computed at the individual level and presented as mean ± SE across participants. Scatter plots show individual-level results for each measure.

To evaluate signal capture in the time domain, characteristic BRO features corresponding to the amplitudes and latencies of the C1 and C2 components were examined and compared between the Textile-EEG and Dry-EEG systems (Figure 5A). Results showed that both C1 and C2 amplitudes were significantly different from pre-blink baseline for the Textile-EEG system (Figure 5B, $t_{8}$ = −7.34, *p* < 0.001 for C1, and $t_{8}$ = 6.51, *p* < 0.001 for C2). These findings suggest that the Textile-EEG system successfully captured characteristic BRO response features consistent with prior literature [13,16,19]. Similar results were found for the Dry-EEG system, with significant amplitude differences compared to baseline for both C1 ($t_{8}$ = −9.64, *p* < 0.001) and C2 components ($t_{8}$ = 6.50, *p* < 0.001) (Figure 5B). Comparison between the two systems showed that no significant differences were found in either amplitude or latency for either component, suggesting that both systems exhibited comparable performance in capturing the characteristic BRO response waveforms.

**Figure 6:**
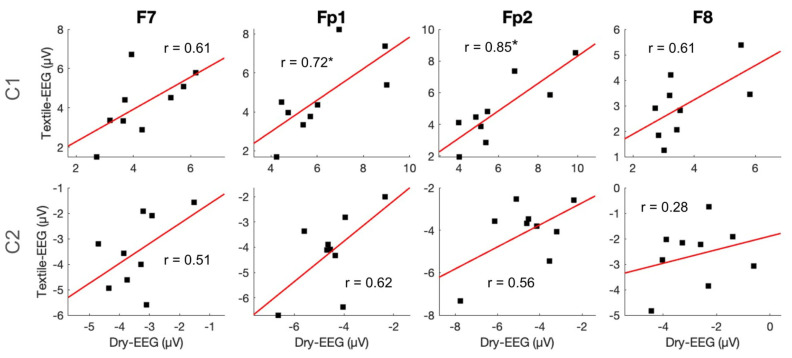
Individual-level time-domain signal capture for amplitudes of C1 and C2 components. Scatter plots show individual-level component amplitudes between the Textile-EEG and Dry-EEG systems, with least squares regression line and the associated correlation coefficients overlaid. * *p* < 0.05.

**Figure 7:**
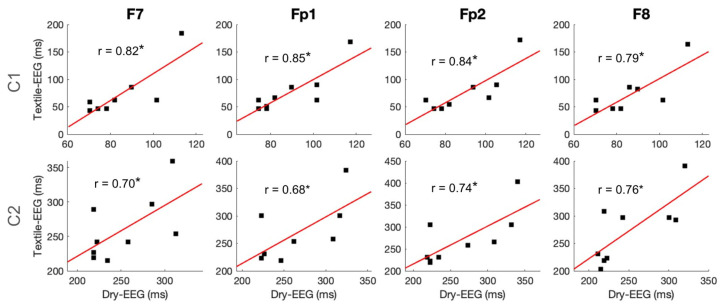
Individual-level time-domain signal capture for latencies of C1 and C2 components. Scatter plots show individual-level component latencies for the Textile-EEG and Dry-EEG systems, with least squares regression line and the associated correlation coefficients overlaid. * *p* < 0.05.

To examine individual-level signal capture in the two technologies, C1 and C2 component amplitudes and latencies were compared between the two technologies using Pearson correlation. Results showed that C1 amplitude exhibited significant high correlations between the Textile-EEG and Dry-EEG systems for the Fp1 and Fp2 channels at the individual level, while the F7 and F8 channels showed a moderate, non-significant correlation (Figure 6, top panel). On the other hand, C2 amplitudes did not show significant correlations between the two technologies (Figure 6, bottom panel). Latency results also showed significant high correlations at the individual level for both C1 and C2 components across most channels (Figure 7). These findings suggest that both the Textile-EEG and Dry-EEG systems captured similar BRO signal characteristics at the individual level for most electrodes in the C1 component, but such correlations were not significant for C2.

### 3.4. Morphological Comparisons

Quantitative morphological comparisons of time-domain BRO responses using ICC showed significant high correlation between the Textile-EEG and Dry-EEG systems for most of the channels (Table 1), suggesting that both technologies captured BRO responses with similar morphologies. Frequency-domain ICC results also showed significant high correlations between the two technologies across several frequency bands. In particular, the low-delta band exhibited significant high correlations in all channels except Fp2, while the mid- and high-delta bands demonstrated significant high correlation in the Fp2 and F7 channels, respectively (Table 1). These results suggest that both the Textile-EEG and Dry-EEG exhibited similar performance in capturing frequency-domain BRO responses.

## 4. Discussion

In this study, we undertook the first investigation of BRO response measurement using a portable EEG system with only four sensors. We compared two EEG systems having the same amplifier hardware, with one system utilizing textile-based electrodes (Textile-EEG), while the other uses dry Ag/AgCl electrodes (Dry-EEG) at the same locations (F7, Fp1, Fp2, F8). Our results showed that BRO responses can be successfully captured using both systems, with similar morphology and signal characteristics in both time and frequency domains. These results demonstrate for the first time that BRO responses are capable of being measured using portable, easy-to-use EEG systems that are conducive to point-of-care deployment, paving the way for future development of a user-friendly platform for BRO measurement in a variety of settings.

### 4.1. Textile-EEG Captures BRO Responses Comparable to Dry-EEG

Both the Textile-EEG and Dry-EEG systems captured raw signal exhibiting ocular artifact at blink latency (Figure 3), with characteristic time- and frequency-domain features in line with prior literature [19]. Data denoising using the TF filter technique in accordance with prior literature successfully removed the ocular artifact for both EEG systems [19,37,38], resulting in cleaned time-domain BRO waveforms that exhibit characteristic post-blink peaks corresponding to the C1 and C2 components (Figure 4 and Figure 5, left panel). Quantitative assessment of artifact removal efficacy also showed greater than 99.5% reduction in ocular signal contribution following artifact removal for both the Textile-EEG and Dry-EEG systems (Figure 3B). Additionally, qualitative evaluation showed the disappearance of signal characteristics consistent with ocular artifact, such as large signal spike at blink latency in the time domain, and signal power concentration at blink latency in the frequency domain (Figure 5). These findings indicate that both the Textile-EEG and Dry-EEG systems functioned correctly in recording the raw signal with anticipated ocular artifact at blink latency, which the TF filter technique subsequently successfully removed for both systems. Both technologies showed comparable performance in capturing the morphological features for both the raw data and cleaned BRO waveforms.

In evaluating BRO response characteristics, quantitative morphological assessment using ICC showed significant high correlations between the Textile-EEG and Dry-EEG systems in both the time and frequency domains in most of the channels (Table 1), suggesting that both systems captured similar BRO response features in the shape of the time-domain waveform as well as the frequency-domain spectral changes. Quantitative comparisons of time-domain BRO characteristics showed that the peak amplitudes for C1 were significantly increased compared to baseline for both technologies, while those of C2 were significantly decreased compared to baseline (Figure 5). These results are also in line with prior literature [13,19], suggesting that both EEG systems detected BRO response features that were real and meaningful. However, there were no differences in amplitude or latency for either C1 or C2 components between the Textile-EEG and Dry-EEG systems, suggesting that both systems exhibited comparable performance in detecting salient BRO characteristics.

There were no significant differences between the two technologies in capturing time-domain features of interest, as the C1 and C2 peaks of both technologies demonstrated similar amplitudes and latencies (Figure 5). These results suggest that both the Textile-EEG and Dry-EEG systems exhibited adequate signal capture capabilities in the time domain. Additional morphological comparisons in the frequency domain showed significant high intraclass correlations between the Dry-EEG and Textile-EEG systems, suggesting that the morphological features of the BRO response were successfully captured by both technologies (Table 1). Interestingly, the polarity of the C1 and C2 BRO components in this study differ from those in prior studies [13,19], which is likely due to the anterior placement of the electrodes in the current study which occupy frontal and prefrontal areas, whereas previous studies focused on the BRO waveforms at posterior electrode locations. Nonetheless, the extracted BRO responses themselves in this study are similar to those of the corresponding electrodes in the prior studies, suggesting that the BRO signals are consistent with prior works.

Individual-level latency measurements for the C1 and C2 post-blink components showed significant high correlations between the Dry-EEG and Textile-EEG systems, indicating that both devices captured waveform features with consistent timing regardless of sensor technology, and the results are robust at the individual level (Figure 7). However, only the C1 component showed significant high correlations in amplitude between the two technologies, while the C2 component amplitudes were non-significantly correlated (Figure 6). These results suggest that, while both devices detected waveform features occurring at similar times, the magnitude of the waveform features were not consistent between the devices. The earlier occurring components such as C1 in BRO responses had previously been postulated to represent sensory processing [12,13], which is a consistent and robust phenomenon both within and across subjects [39]. On the other hand, the later components such as C2 are believed to represent higher-level cognitive processing [13,17], which tends to have greater variability across and within individuals [40]. As such, the high correlation of the C1 component amplitude between the two EEG systems is in line with the robustness of this component as an earlier sensory process within the BRO phenomenon, whereas the C2 component’s reduced consistency between EEG systems may be associated with its potential higher variability as a later-occurring feature of the BRO response.

Another contributing factor to the reduced correlation in C2 component amplitudes may be the spatial location of the electrodes used, as prior studies demonstrated that BRO responses under resting conditions activate the dorsal and ventral visual processing streams spanning the bilateral occipital, posterior parietal, and inferior temporal regions, along with the precuneus in the medial posterior parietal cortex [1]. As such, previous BRO studies have often focused on posterior electrode locations due to the proximity of these electrodes to the precuneus and other posterior regions of the brain as cortical generators of the BRO signal [13,19]. Although the frontal electrodes employed in this study are located farther away from the precuneus, these electrodes were selected based on their positions within the hairless regions of the scalp. Given that achieving adequate electrode-skin contact through hair on the scalp is a critical obstacle in the design and application of textile-based electrodes [41], the use of frontal electrodes that enable direct contact with the skin without electrolytic gel confers a crucial advantage to the Textile-EEG system used in this study. Nevertheless, the frontal electrodes may not have detected BRO effects that were as strong as would have been possible with posterior electrodes, which may also have contributed to the increased variability and reduced consistency in the C2 component amplitude measured using the two EEG systems. Nonetheless, both Textile-EEG and Dry-EEG successfully detected the presence of the BRO phenomenon with consistent features in line with prior literature [13,19], and demonstrated high consistency in component timing between the two systems (Figure 7). These findings suggest that both devices performed comparably well in capturing the BRO response despite the anterior electrode locations. Further studies are needed to investigate BRO signal capture using textile-based electrodes located in more posterior positions.

### 4.2. Textile-EEG Represents Advance in Portable BRO Applications

Conventional wet and dry electrodes both comprise Ag/Ag-Cl material, but wet electrodes also utilize electrolytic gel at the electrode–skin interface in order to enhance conductivity. Although wet electrodes provide better signal quality over short durations, the use of gel also has several disadvantages, including participant discomfort [42], irritation or allergic reactions in the skin [42], the drying of gel over time that compromises signal quality in long-duration recordings [43], and a requirement for abrasive skin preparation to remove outer layers of dead skin cells and expose inner, more conductive layers of the epidermis [44]. Prior studies of BRO responses have employed wet-electrode EEG systems, which are known to have lower impedance and better signal quality compared to dry electrode systems. Nonetheless, the particular dry-electrode EEG system used in the current study has previously been demonstrated to exhibit comparable performance relative to wet-electrode EEG systems in detecting sensorimotor neural responses for brain computer interface applications [33], suggesting that these systems are capable of capturing neural responses with robust performance despite their higher impedance compared to wet-electrode systems. As such, the dry electrode system may serve as an ‘intermediate benchmark’ in evaluating signal quality for textile-based EEG in mobile and portable applications, and was thus employed as a signal quality comparison for BRO measurement in the current study. Our results in this study demonstrate that Textile-EEG exhibited comparable performance relative to the Dry-EEG system in BRO signal capture, providing further support for the future development of a portable and easy-to-use BRO measurement technology based on textile electrodes—which would also be more comfortable to wear compared to dry-electrode systems.

Traditional EEG data acquisition systems are large, bulky, require long testing setup times, and are not comfortable to wear [45]. The use of textile-based electrodes for EEG systems began to gain traction in the early 2000s, when researchers started investigating textile materials as a more comfortable alternative to conventional Ag/Ag-Cl electrodes [46]. The main goal in textile electrode development at that time was to create soft and stretchable EEG sensors that were capable of adapting to the dynamic shape of human skin in order to accommodate motions such as bending, folding, and twisting [43,47]. The resulting designs were capable of accommodating strains of up to 30% [48,49,50,51]. Since that time, the field has grown significantly, with the potential of greater user acceptance and decreased costs [52,53].

The most commonly used conductive materials for textile-based EEG systems include metals, conductive polymers, and carbon allotropes such as graphene or carbon nanotubes, which are integrated into non-conductive fabric through different fabrication techniques [43]. There are many conventional fabric manufacturing approaches that provide easy and inexpensive methods for producing textile-based electrodes, such as embroidering, sewing, knitting, and weaving [54]. In addition, techniques like dip-coating [55], chemical polymerization [56], electroplating [57,58], physical vapor deposition (PVD) [59,60], and printing methods [61] can be applied onto finished, non-conductive textiles in order to coat the surface of the textiles to enable conductivity. Several studies have reported textile-based EEG systems developed using these materials and fabrication techniques. For instance, one study successfully demonstrated EEG data collection using Ag/AgCl-coated threads integrated into a nylon headband [62]. Another study developed a textile-based EEG system using a Ag-powder/fluoropolymer-based nanocomposite ink fabricated into two-layers of textiles by jet-printing, with electrodes placed across the forehead and behind the ear in hairless regions [63]. However, despite their success, these prior studies using textile-based EEG systems all demonstrated only resting-state EEG data collection, with quantitative EEG (qEEG) analysis that focuses on signal power in different frequency bands [64]. Such approach cannot elucidate the rapid temporal dynamics of neural processing, and none of the prior studies have demonstrated successful EEG measurement using event-related experimental paradigms such as event-related potentials (ERPs) that can capture neural processing occurring on a millisecond scale.

The current study is the first time that BRO responses have been measured using a textile-based EEG system. The portable EEG hardware and textile-based form factor facilitate data collections not only in lab settings, but also in mobile applications outside the lab. Relative to existing consumer-grade EEG devices currently available on the market which are based on rigid materials and fixed form factor design, the Textile-EEG device represents a major breakthrough in creating EEG technologies that enable longer wear and enhanced ergonomic experiences. Moreover, contrary to traditional EEG-based approaches such as ERPs that require external sensory stimuli such as images or sounds to elicit specific brain responses for evaluation, the BRO phenomenon corresponds to neural processing of new visual input that appears after the naturally occurring activity of blinking. As such, BRO responses provide naturalistic assessments of endogenous, event-related brain responses time-locked to blinking—without requiring external sensory stimuli. In light of this, the current study provides the first demonstration of the capture of event-related brainwave responses using a textile-based EEG system. Crucially, given the simplicity and ubiquity of spontaneous blinking in everyday life, the successful capture of blink-related brainwave responses using textile-based EEG creates a new approach that leverages blinking as an easy-to-access window into the brain—enabling naturalistic assessments of brain function in a variety of settings through devices that are easy to use and comfortable to wear.

### 4.3. Study Limitations

Several limitations should be noted in this study. Given the small sample size of 10 participants, the findings provide promising preliminary evidence in demonstrating the feasibility of measuring BRO responses using textile-based EEG systems. Future studies are needed to confirm these findings using larger samples. In addition, the experimental paradigm utilized an eyes-open resting-state task, which does not provide information about BRO responses under other task states. Future studies are needed to validate the recording of BRO responses under diverse experimental conditions in order to demonstrate the feasibility of textile-based EEG for BRO response capture in a variety of situations. Finally, the current study utilized EEG data collected within research settings, and future studies should investigate the potential of BRO measurement in point-of-care settings to demonstrate the practicality and feasibility of widespread deployment.

## 5. Conclusions

This study investigated BRO measurement using low-density EEG systems with either textile-based or dry Ag/AgCl electrodes. Our results showed that BRO responses can be successfully captured using only 4 sensors, and that both systems demonstrated comparable performance in the signal capture of BRO responses. This is the first time that BRO responses have been captured using portable, low-density EEG systems with only four sensors, and our results provide promising initial evidence towards the feasibility of rapid and easy-to-apply BRO measurement using textile-based electrodes to enhance user friendliness and enable future point-of-care deployment in a variety of settings.

## Figures and Tables

**Table 1:** ICC coefficients showing similarities at an individual level between the Textile-EEG and Dry-EEG data in time and frequency domains. Results are presented as mean ICC value ± SE across all subjects. * *p* < 0.05.

	F7	Fp1	Fp2	F8
Time Domain	0.73 ± 0.06 *	0.77 ± 0.05 *	0.75 ± 0.04 *	0.59 ± 0.08
Low Delta (0.5–1.5 Hz)	0.89 ± 0.05 *	0.95 ± 0.02 *	0.84 ± 0.07	0.71 ± 0.11 *
Mid-Delta (1.5–3.5 Hz)	0.82 ± 0.07	0.84 ± 0.07	0.87 ± 0.04 *	0.71 ± 0.10
High Delta (3.5–4.5 Hz)	0.83 ± 0.04 *	0.78 ± 0.05	0.77 ± 0.03	0.77 ± 0.04

## Data Availability

Data for this study are publicly available at https://www.frontiersin.org/articles/10.3389/fnhum.2023.1135153/full#supplementary-material. (accessed on 17 June 2025).

## References

[1] Liu C.C., Hajra S.G., Cheung T.P.L., Song X., D’Arcy R.C.N. (2017). Spontaneous blinks activate the precuneus: Characterizing blink-related oscillations using magnetoencephalography. Front. Hum. Neurosci..

[2] Bonfiglio L., Olcese U., Rossi B., Frisoli A., Arrighi P., Greco G., Carozzo S., Andre P., Bergamasco M., Carboncini M.C. (2013). Cortical source of blink-related delta oscillations and their correlation with levels of consciousness. Hum. Brain Mapp..

[3] Nagahamaa Y., Okadab T., Katsumic Y., Hayashic T., Yamauchic H., Sawamotoa N., Tomaa K., Nakamuraa K., Hanakawaa T., Konishib J. (1999). Transient neural activity in the medial superior frontal gyrus and precuneus time locked with attention shift between object features. NeuroImage.

[4] Malouin F., Richards C.L., Jackson P.L., Dumas F., Doyon J. (2003). Brain activations during motor imagery of locomotor-related tasks: A PET study. Hum. Brain Mapp..

[5] Wenderoth N., Debaere F., Sunaert S., Swinnen S.P. (2005). The role of anterior cingulate cortex and precuneus in the coordination of motor behaviour. Eur. J. Neurosci..

[6] Shallice T., Fletcher P., Frith C.D., Grasby P., Frackowiak R.S.J., Dolan R.J. (1994). Brain regions associated with acquisition and retrieval of verbal episodic memory. Nature.

[7] Gilboa A., Winocur G., Grady C.L., Hevenor S.J., Moscovitch M. (2004). Remembering our past: Functional neuroanatomy of recollection of recent and very remote personal events. Cereb. Cortex.

[8] Lundstrom B.N., Ingvar M., Petersson K.M. (2005). The role of precuneus and left inferior frontal cortex during source memory episodic retrieval. NeuroImage.

[9] Kircher T.T.J., Senior C., Phillips M.L., Benson P.J., Bullmore E.T., Brammer M., Simmons A., Williams S.C., Bartels M., David A.S. (2000). Towards a functional neuroanatomy of self processing: Effects of faces and words. Cogn. Brain Res..

[10] Farrer C., Frith C. (2002). Experiencing oneself vs another person as being the cause of an action: The neural correlates of the experience of agency. NeuroImage.

[11] Kjaer T.W., Nowak M., Lou H.C. (2002). Reflective self-awareness and conscious states: PET evidence for a common midline parietofrontal core. NeuroImage.

[12] Liu C.C., Hajra S.G., Song X., Doesburg S.M., Cheung T.P.L., D’Arcy R.C.N. (2018). Cognitive loading via mental arithmetic modulates effects of blink-related oscillations on precuneus and ventral attention network regions. Hum. Brain Mapp..

[13] Liu C.C., Hajra S.G., Pawlowski G., Fickling S.D., Song X., D’Arcy R.C.N. (2020). Differential neural processing of spontaneous blinking under visual and auditory sensory environments: An EEG investigation of blink-related oscillations. NeuroImage.

[14] Hajra S.G., Liu C.C., Law A. Neural responses to spontaneous blinking capture differences in working memory load: Assessing blink related oscillations with N-back task. Proceedings of the Interntional Neuroergonomics Conference.

[15] Page C., Liu C.C., Meltzer J., Hajra S.G. (2024). Blink-Related Oscillations Provide Naturalistic Assessments of Brain Function and Cognitive Workload within Complex Real-World Multitasking Environments. Sensors.

[16] Ziccardi A., Van Benthem K., Liu C.C., Herdman C.M., Hajra S.G. (2024). Towards ubiquitous and nonintrusive measurements of brain function in the real world: Assessing blink-related oscillations during simulated flight using portable low-cost EEG. Front. Neurosci..

[17] Hajra S.G., Meltzer J.A., Keerthi P., Pappas C., Sekuler A.B., Liu C.C., Cam-CAN Group (2025). Spontaneous blinking and brain health in aging: Large-scale evaluation of blink-related oscillations across the lifespan. Front. Aging Neurosci..

[18] Sattari S., Kenny R., Liu C.C., Hajra S.G., Dumont G.A., Virji-Babul N. (2023). Blink-related EEG oscillations are neurophysiological indicators of subconcussive head impacts in female soccer players: A preliminary study. Front. Hum. Neurosci..

[19] Liu C.C., Hajra S.G., Fickling S.D., Pawlowski G., Song X., D’Arcy R.C.N. (2019). Novel Signal Processing Technique for Capture and Isolation of Blink-Related Oscillations Using a Low-Density Electrode Array for Bedside Evaluation of Consciousness. IEEE Trans. Biomed. Eng..

[20] Lopez-Gordo M.A., Sanchez-Morillo D., Valle F.P. (2014). Dry EEG Electrodes. Sensors.

[21] Leleux P., Badier J., Rivnay J., Bénar C., Hervé T., Chauvel P., Malliaras G.G. (2013). Conducting Polymer Electrodes for Electroencephalography. Adv. Healthc. Mater..

[22] Posada-Quintero H.F., Reyes B.A., Burnham K., Pennace J., Chon K.H. (2015). Low Impedance Carbon Adhesive Electrodes with Long Shelf Life. Ann. Biomed. Eng..

[23] Chi M., Zhao J., Dong Y., Wang X. (2019). Flexible Carbon Nanotube-Based Polymer Electrode for Long-Term Electrocardiographic Recording. Materials.

[24] Ko L.W., Su C.H., Liao P.L., Liang J.T., Tseng Y.H., Chen S.H. (2021). Flexible graphene/GO electrode for gel-free EEG. J. Neural Eng..

[25] Vidhya C.M., Maithani Y., Singh J.P. (2023). Recent Advances and Challenges in Textile Electrodes for Wearable Biopotential Signal Monitoring: A Comprehensive Review. Biosensors.

[26] Fu Y., Zhao J., Dong Y., Wang X. (2020). Dry Electrodes for Human Bioelectrical Signal Monitoring. Sensors.

[27] López-Larraz E., Escolano C., Robledo-Menéndez A., Morlas L., Alda A., Minguez J. (2023). A garment that measures brain activity: Proof of concept of an EEG sensor layer fully implemented with smart textiles. Front. Hum. Neurosci..

[28] Xiao X., Pirbhulal S., Dong K., Wu W., Mei X. (2017). Performance Evaluation of Plain Weave and Honeycomb Weave Electrodes for Human ECG Monitoring. J. Sens..

[29] Maithani Y., Choudhuri B., Mehta B.R., Singh J.P. (2020). A comprehensive review of the fabrication and performance evaluation of dry electrodes for long-term ECG monitoring. Model. Anal. Act. Biopotential Signals Healthc..

[30] Bandara V., Nanayakkara A. (2020). A low-cost, portable biopotential monitoring system to study electrical activities of the human brain and body. Eur. J. Phys..

[31] Löfhede J., Seoane F., Thordstein M. (2012). Textile electrodes for EEG recording-A pilot study. Sensors.

[32] Golparvar A., Ozturk O., Yapici M.K. Gel-Free Wearable Electroencephalography (EEG) with Soft Graphene Textiles. Proceedings of the IEEE Sensors.

[33] Schwarz A., Escolano C., Montesano L., Müller-Putz G.R. (2020). Analyzing and Decoding Natural Reach-and-Grasp Actions Using Gel, Water and Dry EEG Systems. Front. Neurosci..

[34] Hajra S.G., Liu C.C., Song X., Fickling S.D., Cheung T.P.L., D’Arcy R.C.N. (2018). Multimodal characterization of the semantic N400 response within a rapid evaluation brain vital sign framework. J. Transl. Med..

[35] McGraw K.O., Wong S.P. (1996). Forming Inferences about Some Intraclass Correlation Coefficients. Psychol. Methods.

[36] Maris E., Oostenveld R. (2007). Nonparametric statistical testing of EEG- and MEG-data. J. Neurosci. Methods.

[37] Hajra S.G., Liu C.C., Fickling S.D., Pawlowski G.M., Song X., D’Arcy R.C.N. (2021). Event related potential signal capture can be enhanced through dynamic snr-weighted channel pooling. Sensors.

[38] Hajra S.G., Gopinath S., Liu C.C., Pawlowski G., Fickling S.D., Song X., D’Arcy R.C. Enabling event-related potential assessments using low-density electrode arrays: A new technique for denoising individual channel EEG data. Proceedings of the IEMTRONICS 2020-International IOT, Electronics and Mechatronics Conference.

[39] Du X., Choa F.-S., Summerfelt A., Rowland L.M., Chiappelli J., Kochunov P., Hong L.E. (2016). N100 as a generic cortical electrophysiological marker based on decomposition of TMS-evoked potentials across five anatomic locations. Exp. Brain Res..

[40] Polich J. (2007). Updating P300: An Integrative Theory of P3a and P3b. Clin. Neurophysiol..

[41] Chan H.L., Kuo P.C., Cheng C.Y., Chen Y.S. (2018). Challenges and Future Perspectives on Electroencephalogram-Based Biometrics in Person Recognition. Front. Neurosci..

[42] Chi Y.M., Jung T.P., Cauwenberghs G. (2010). Dry-contact and noncontact biopotential electrodes: Methodological review. IEEE Rev. Biomed. Eng..

[43] Acar G., Ozturk O., Golparvar A.J., Elboshra T.A., Böhringer K., Yapici M.K. (2019). Wearable and flexible textile electrodes for biopotential signal monitoring: A review. Electronics.

[44] Huigen E. (2001). Noise Characteristics of Surface Electrodes. Master’s Thesis.

[45] Carneiro M.R., de Almeida A.T., Tavakoli M. (2020). Wearable and Comfortable e-Textile Headband for Long-Term Acquisition of Forehead EEG Signals. IEEE Sensors J..

[46] Hughes-Riley T., Dias T., Cork C. (2018). A Historical Review of the Development of Electronic Textiles. Fibers.

[47] Cömert A., Honkala M., Hyttinen J. (2013). Effect of pressure and padding on motion artifact of textile electrodes. Biomed. Eng. Online.

[48] Nag A., Mukhopadhyay S.C., Kosel J. (2017). Wearable Flexible Sensors: A Review. IEEE Sens. J..

[49] Shao Y., Hu H., Visell Y. (2020). A Wearable Tactile Sensor Array for Large Area Remote Vibration Sensing in the Hand. IEEE Sens. J..

[50] Lopes P.A., Gomes D.V., Marques D.G., Faia P., Góis J., Patrício T.F., Coelho J., Serra A., de Almeida A.T., Majidi C. (2019). Soft Bioelectronic Stickers: Selection and Evaluation of Skin-Interfacing Electrodes. Adv. Healthc. Mater..

[51] Lopes P.A., Paisana H., De Almeida A.T., Majidi C., Tavakoli M. (2018). Hydroprinted Electronics: Ultrathin Stretchable Ag–In–Ga E-Skin for Bioelectronics and Human–Machine Interaction. ACS Appl. Mater. Interfaces.

[52] Ismar E., Bahadir S.K., Kalaoglu F., Koncar V. (2020). Futuristic Clothes: Electronic Textiles and Wearable Technologies. Glob. Chall..

[53] Heo J.S., Eom J., Kim Y.H., Park S.K. (2018). Recent Progress of Textile-Based Wearable Electronics: A Comprehensive Review of Materials, Devices, and Applications. Small.

[54] Stoppa M., Chiolerio A. (2014). Wearable electronics and smart textiles: A critical review. Sensors.

[55] Yapici M.K., Alkhidir T., Samad Y.A., Liao K. (2015). Graphene-clad textile electrodes for electrocardiogram monitoring. Sensors Actuators B: Chem..

[56] Zhou Y., Ding X., Zhang J., Duan Y., Hu J., Yang X. (2014). Fabrication of conductive fabric as textile electrode for ECG monitoring. Fibers Polym..

[57] Qin H., Li J., He B., Sun J., Li L., Qian L. (2018). Novel Wearable Electrodes Based on Conductive Chitosan Fabrics and Their Application in Smart Garments. Materials.

[58] Lee S.H., Jung S.M., Lee C.K., Jeong K.S., Cho G., Yoo S.K. (2009). Wearable ECG Monitoring System Using Conductive Fabrics and Active Electrodes. Lecture Notes in Computer Science (including subseries Lecture Notes in Artificial Intelligence and Lecture Notes in Bioinformatics), Proceedings of the International Conference on Human-Computer Interaction, San Diego, CA, USA, 19–24 July 2018.

[59] Cho G., Jeong K., Paik M.J., Kwun Y., Sung M. (2011). Performance evaluation of textile-based electrodes and motion sensors for smart clothing. IEEE Sens. J..

[60] Alzaidi A., Zhang L., Bajwa H. Smart textiles based wireless ECG system. Proceedings of the 2012 IEEE Long Island Systems, Applications and Technology Conference, LISAT 2012.

[61] Takamatsu S., Lonjaret T., Crisp D., Badier J.-M., Malliaras G.G., Ismailova E. (2015). Direct patterning of organic conductors on knitted textiles for long-term electrocardiography. Sci. Rep..

[62] Fleury A., Alizadeh M., Stefan G., Chau T. Toward fabric-based EEG access technologies: Seamless knit electrodes for a portable brain-computer interface. Proceedings of the 2017 IEEE Life Sciences Conference, LSC 2017.

[63] La T., Qiu S., Scott D.K., Bakhtiari R., Kuziek J.W.P., Mathewson K.E., Rieger J., Chung H. (2018). Two-Layered and Stretchable e-Textile Patches for Wearable Healthcare Electronics. Adv. Healthc. Mater..

[64] Popa L.L., Dragos H., Pantelemon C., Rosu O.V., Strilciuc S. (2020). The Role of Quantitative EEG in the Diagnosis of Neuropsychiatric Disorders. J. Med. Life.

